# Later-life migration and depression among older adults in India: examining the role of functional limitations

**DOI:** 10.1038/s41598-025-14489-9

**Published:** 2025-10-22

**Authors:** Bittu Mandal, Kalandi Charan Pradhan

**Affiliations:** School of Humanities and Social Sciences, Indian Institute of Technology Indore, Khandwa Road, Simrol, 453552 Indore India

**Keywords:** Health, Migration, Depression, Functional limitation, India, Diseases, Risk factors

## Abstract

The prevalence of depression is increasing in low- and middle-income countries (LMICs) such as India; migration is often associated with this rise. Nevertheless, there is limited knowledge about the moderating effect of functional limitations in the association between age at migration and depression in the middle and old age population. This study aimed to examine how functional limitations moderate the association between age at migration and depression in older adults in India. We utilized Longitudinal Aging Study in India data, comprising a sample of 61,855 adults aged 45 and above. Depression was the outcome variable. We employed negative binomial regression and interaction analyses to fulfill the study objective. Migrants (9.94) reported higher mean depressive scores than non-migrants (9.44). Migrants with Activities of Daily Living limitations (ADL) who migrated at a younger age (< 20 years) had 1.12 times higher risk, those at the age of 20–39 had 1.13 times higher risk, and those who migrated at the age of 40–59 had 1.15 times higher risk of depression than their non-migrant counterparts without any ADL limitations. The findings suggest that migrating at older ages comes with a higher psychological risk. Functional limitations significantly amplify the development of depression among migrants. The results advocate specific interventions aimed at reducing psychological stress among migrants who migrate at later ages and have functional limitations.

**Supplementary Information:**

The online version contains supplementary material available at 10.1038/s41598-025-14489-9.

## Introduction

Migration is a complex and multifaceted phenomenon that has become increasingly prevalent in the globalized world^1^. Individuals migrate for various reasons, including economic opportunities, education, political asylum, and family reunification^2–6^. Migration can lead to improved health outcomes, as migrants often gain access to better healthcare systems and benefit from increased economic affluence in their destination places, especially those who are moving from resource constraint to prosperous settings, enabling them to utilize quality medical services and maintain healthier lifestyles^7,8^. This positive aspect underscores the potential for migration to enhance well-being and quality of life. However, migration also presents challenges, particularly in the realm of mental health^9,10^. One intriguing aspect that researchers have begun to delve into is the relationship between the age at which individuals migrate and their susceptibility to depression^11^. Understanding the impact of age at migration on mental health, specifically depression, is crucial for several reasons. First, the process of cultural adaptation and acculturation can vary significantly depending on the developmental stage at which migration occurs^12^. Second, individuals who migrate during their formative years may face unique challenges related to identity formation, social integration, and coping mechanisms^12,13^. However, the stress associated with the process of migration and cultural adaptation could expose migrants to a greater likelihood of experiencing depressive symptoms compared to individuals born in their native places^14,15^.

Existing literature on migration and mental health has primarily focused on acculturation stress, social isolation, and discrimination as key factors influencing mental well-being^16–20^. However, the role of age at migration in shaping these experiences and outcomes has gained attention only recently. Studies have shown that individuals who migrate during adolescence or early adulthood may grapple with identity conflicts, language barriers, and academic challenges, which can contribute to increased vulnerability to depression^21^. On the other hand, those who migrate later in life may face issues related to social isolation, loss of social networks, and difficulties adapting to a new cultural environment^22,23^.

To understand the association between age at migration and depression, *Life Course Theory* offers a valuable framework^24^. This theory emphasizes that life experiences, including migration, must be examined within the context of timing, transitions, and trajectories over the individual’s lifespan. According to *Life Course Theory*, the age at which migration occurs is crucial because it shapes exposure to both protective and risk factors during key developmental stages. Early-life migration, particularly during childhood and adolescence, can disrupt critical developmental processes such as identity formation, schooling, and peer integration, potentially leading to long-term vulnerabilities to poor mental health, including depression. On the other hand, migration at a later age, particularly during adulthood or even after that, can be more stressful. Older migrants may face more difficulties in adjusting to the new cultural setting because their habits, values, and ways of life are already formed. They may also find it harder to learn the local language, find suitable jobs, or build strong social circles. Such difficulties in adjustment can increase their risk of isolation, cultural stress, and ultimately depression. *Life Course Theory* offers a detailed perspective on migration that it is not just a one-time event, but a process that affects overall wellbeing and mental health. The age at which a person migrates decides how well they can cope with changes, which in turn affects their mental health in the long run.

Given this understanding of migration as a process influencing long-term mental health, it is crucial to examine how specific factors, such as physical limitations, interact with the timing of migration to influence outcomes like depression. A limited study has analyzed the moderating effect of physical limitation on the association between age at migration and depression. A number of previous studies indicate that physical constraints tend to moderate the direction and magnitude of the relationship between age at migration and depression^25^. The concept of moderation introduces the idea that the relationship between age at migration and depression is not uniform for all individuals^26^. Cariello and colleagues examined the presence of physical limitations as a moderating factor that can either amplify or mitigate the impact of migration on mental health, particularly depression^27^. Physical limitations may encompass a range of health issues, including chronic illnesses, disabilities, or other health-related constraints that affect an individual’s daily functioning^28^. These limitations can intensify the challenges associated with migration, as individuals with physical constraints may find it more difficult to adapt to new environments, engage in social activities, or access necessary healthcare services^29^. In the context of the moderating effect, the presence of physical limitations could exacerbate the negative impact of migration on mental health. For instance, individuals migrating at an older age who also experience physical limitations may face increased difficulties in establishing social connections, accessing healthcare, and coping with the stressors of adapting to a new culture^30^. These challenges may contribute to a higher likelihood of experiencing depression.

Few studies on the Indian population have investigated migration, physical health, and functional limitations and its association with depressive symptoms^31,32^. However, the existing pieces of literature are not enough to depict the scenario for the entire India. Moreover, there has been no study focusing on the association of age at migration with depressive symptoms and considering the role of physical limitation. Thus, the present study has two primary aims: (i) to examine whether migration at later ages is associated with elevated depression and (ii) to examine the role of physical limitations in moderating the association between age at migration and depressive symptoms among middle-aged and older adults in India.

## Methods

### Data

**Fig.1:**
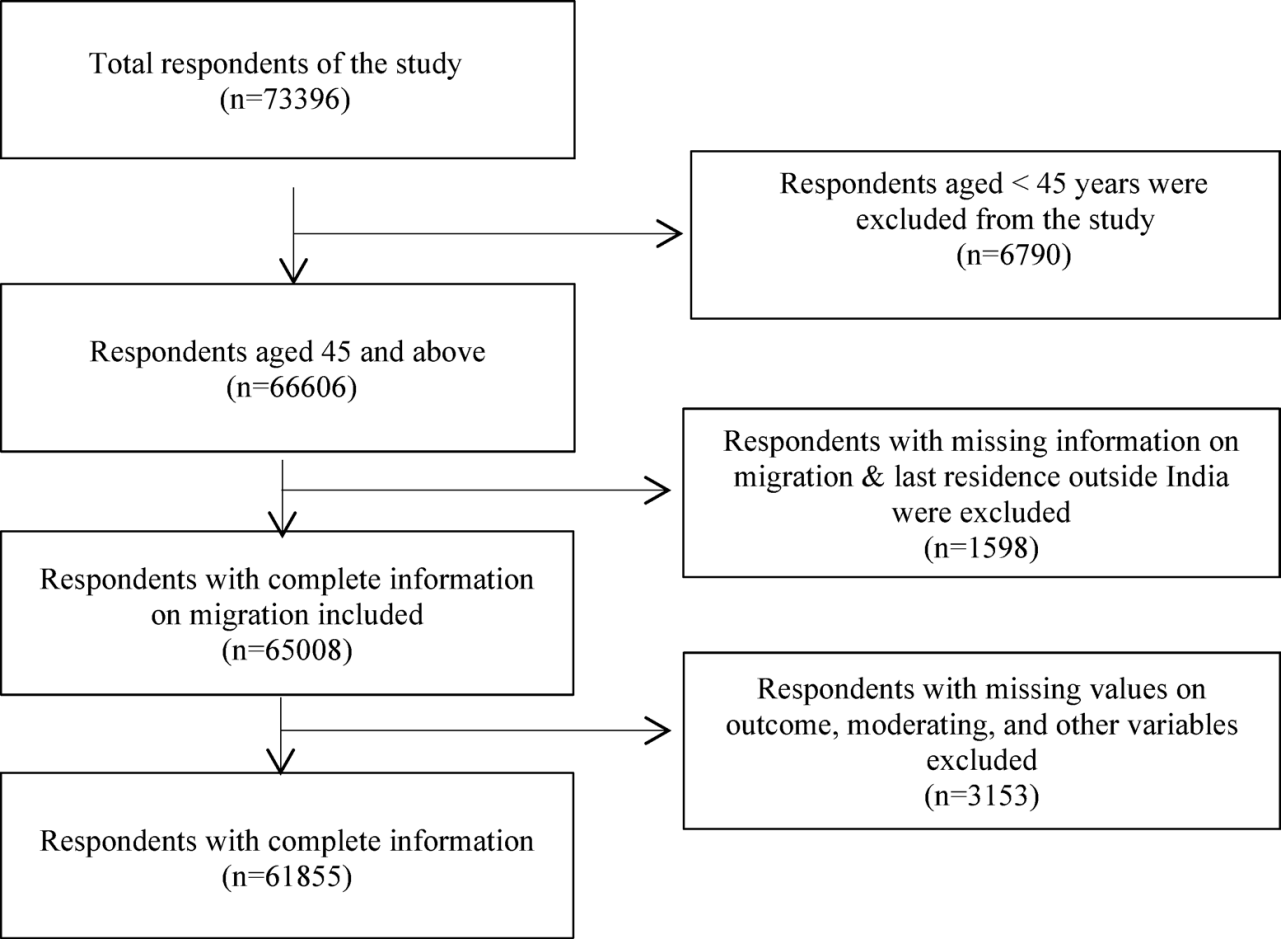
Flowchart of sample selection.

LASI, the Longitudinal Ageing Study in India, represents comprehensive national data focusing on the health, economic, and social determinants associated with population ageing in the country. The primary objective of the LASI survey was to investigate the health status and social and economic well-being of the elderly population aged 45 and above, along with their spouses, regardless of age^33^ (Fig. 1).

#### Sample size calculation and sampling procedure

The LASI sampling frame included only the community-dwelling population. The survey employed a multistage stratified area probability cluster sampling design, distinguishing between rural and urban areas. Within each state, LASI Wave 1 adopted a three-stage sampling design for rural regions and a four-stage sampling design for urban areas. The initial stage involved the selection of Primary Sampling Units (PSUs), i.e., sub-districts (Tehsils/Talukas), followed by selecting villages in rural areas and wards in urban areas in the chosen PSUs. In rural areas, the third stage involved selecting households from the chosen villages, while in urban areas, one Census Enumeration Block (CEB) was randomly selected in each urban area during the third stage. Subsequently, households were chosen from this selected CEB in the fourth stage. The overall individual response rate was 87.3%, and the overall household response rate was 95.8%. The detailed survey methodology, including sample selection and data collection information, is available in the published survey report and elsewhere^33–35^. LASI received approval from the Ethics Committee of the Indian Council of Medical Research (ICMR). The study followed established guidelines and regulations to ensure ethical compliance. Informed consent was taken from participants, and all procedures adhered to ethical standards.

#### Current sample

The sample included in this study comprised 61,855 middle-aged and older adults, with 28,801 males and 33,054 females. The mean age of the total sample was 59, with a standard deviation of ± 10.50 years. The sample consisted of 27,010 migrants and 34,845 non-migrants. The detailed sample selection procedure is provided in Fig. 2.

#### Conceptual framework

**Fig. 2:**
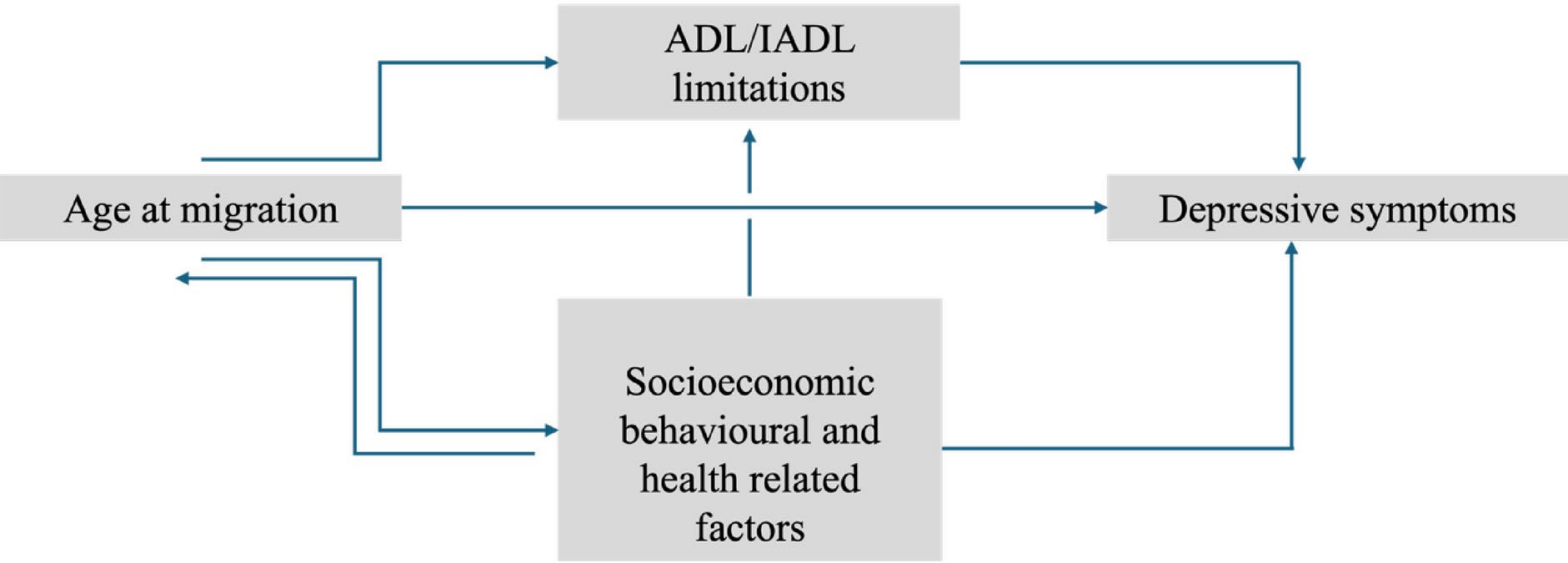
Conceptual framework.

### Measures

#### Outcome variable

##### Depressive symptoms

A modified version of the Centre for Epidemiologic Studies Depression Scale (CES-D), consisting of a shortened 10-item questionnaire with four response items, was employed in the LASI to evaluate depressive symptoms^36^. This scale included ten items comprising seven negative symptoms (trouble concentrating, feeling depressed, low energy, fear of something, feeling alone, bothered by things, and everything is an effort) and three positive symptoms (feeling happy, hopeful, and satisfied). Response options encompassed “rarely or never” (< 1 day), “sometimes” (1 or 2 days), “often” (3 or 4 days), and “most or all of the time” (5–7 days) in the week preceding the interview. Positive symptom scores were reverse-coded. The overall CES-D score ranged from 0 to 30, with a higher score signifying elevated depressive symptoms. The study reported a coefficient alpha of 0.71 for the CES-D, a reliability measures consistent with values from previous studies^37,38^. Table A2 in the appendix provides all the questions that were used to access CES-D scores.

#### Moderating variable

Migration is a complex and often stressful life transition that can significantly impact mental health, particularly increasing the risk of depression. However, not all migrants experience depression to the same extent, suggesting that certain factors may moderate this relationship. One such factor is functional limitation, which encompasses physical impairments that restrict daily activities and reduce independence. Research has consistently shown that functional limitations are strongly associated with higher levels of depression, as they exacerbate psychological distress by limiting mobility, increasing dependency, and reducing social participation^39–41^​​​​. Prior studies have demonstrated that limitations in both basic activities of daily living (ADL) and Instrumental Activities of Daily Living (IADL) independently increase the risk of depression​, and that depression and functional decline can form a bidirectional cycle, where each worsens the other over time​. Given this evidence, functional limitation serves as a critical moderator in understanding the migration-depression relationship, allowing us to assess whether migrants with physical impairments experience disproportionately higher levels of depression. Below, we have provided a detailed description of ADL and IADL, which are key measures of functional limitations.

##### ADL

ADL is a term used to refer to normal daily self-care activities such as movement in bed, changing position from sitting to standing, feeding, bathing, dressing, grooming, and personal hygiene (Table A3). The ability or inability to perform ADLs is used to measure a person’s functional status, especially in the case of people with disabilities and older adults. For this study, ADL was dichotomized as “1” for the older adults who have 1 + ADL limitation; otherwise, it was “0”^42^.

##### IADL

Difficulties or impairments in executing tasks necessary for independent living but not essential for basic self-care are referred to as IADL limitations. Unlike ADL, which incorporates fundamental self-care tasks like bathing and eating, IADLs involve more complex activities such as managing finances, cooking, shopping, using transportation, and handling medications. The assessment of IADL limitations is a common practice in healthcare and gerontology to evaluate an individual’s functional independence and overall ability to live independently in the community. Identifying the presence of IADL limitations offers insights into a person’s level of functioning and serves as a tool to assess the necessity for support or assistance in specific daily activities^29,43^. Please refer to Table A4 for the details of the instruments used in the IADL.

#### Main explanatory variable

#### Migration status and age at migration

According to the UN Manual VI definition, migration is defined as a move from one migration-defining area to another that was made during a given migration interval and involved a change of residence^44^. Additionally, following the Census of India, the concept of the Place of Last Residence is employed to identify migrants; if the location where an individual is enumerated during the census/survey differs from their most recent residence, they are categorized as a migrant^45^. The place of last residence is particularly important since it captures the latest relocation of a person. Moreover, it also accounts for return migration^46^. The definition of migrants in this study is based on their place of last residence. A migrant is someone whose current place of residence differs from their usual place of residence, involving the crossing of any administrative boundary and a permanent change of residence. However, individuals whose last residence was outside India (immigrants) are not excluded from the thesis, as this thesis focuses exclusively on internal migration.

The age at migration is defined as the age at which a migrant made their most recent move to their current location. It is calculated by subtracting the duration of residence at the current place of enumeration from the migrant’s current age at the time of survey. Age at migration was further classified into four categories: migrated before age 20, migrated at age 20–39, migrated at age 40–59, and migrated in later life (age 60 or older)^47–49^.

#### Covariates

Age, sex, marital status, living arrangement, employment status, MPCE, food insecurity, religion, caste, residence, physical activity, smoking, alcohol, self-rated health, multimorbidity and cognitive impairment were considered in this study^50–53^. A detailed list of covariates is provided in Table A1 in the appendix.

### Econometric analysis

In this study, descriptive statistics were reported, and bivariate analysis was performed to assess the mean depressive symptoms overall key explanatory variables. Since our focus is on depressive scores (the score of depressive symptoms ranging from 0 to 27) as the dependent variable, which is expressed as integer numbers, we needed to use models suitable for count data^54^. The Poisson model is widely used for this type of data specification.

1 $$\:{f}_{y}\left(y;\mu\:\right)={e}^{-\mu\:}*\frac{\mu\:}{y!}$$

In this context, $\:y$ denotes a non-negative number, indicating the occurrences (depression), and $\:\mu\:$ stands for the expected number of occurrences, often referred to as the ‘intensity or rate parameter.’ The Poisson distribution has a single parameter, and it assumes equality between the mean and the variance, known as equidispersion.

2 $$\:{\mu\:}_{i}=\text{exp}\left({x}_{i}\beta\:\right)=E\left[{y}_{i}|{x}_{i}\right]=Var\left[{y}_{i}|{x}_{i}\right]$$

where $\:x$ is the vector of explanatory variables.

Nonetheless, the assumption of equal dispersion poses a significant drawback for the Poisson model. In many cases, actual data tend to show overdispersion, where the variance exceeds the mean, in this study the mean of the depressive scores was 9.56 where the variance was 16.37. In such instances, the traditional Poisson model can lead to significant inaccuracies in estimating parameters^55^.

The traditional way to deal with overdispersion is to use mixture models. the mean in Eq. (2) is replaced by:

3 $$\:{\mu\:}_{i}^{*}=xp\left({x}_{i}\beta\:\right)\text{e}\text{x}\text{p}\left({\epsilon\:}_{i}\right)$$

The negative binomial model is a type of mixture model where the $\:\text{e}\text{x}\text{p}\left({\epsilon\:}_{i}\right)$ is assumed to be selected from a gamma distribution. This results in a probability density function:

4 $$\:\text{Pr}\left(y=M=x\right)=\frac{{\Gamma\:}(\gamma\:+{\alpha\:}^{-1})}{\gamma\:!{\Gamma\:}\left({\alpha\:}^{-1}\right)}\:{\left[\frac{{\alpha\:}^{-1}}{{\alpha\:}^{-1}+\mu\:}\right]}^{{\alpha\:}^{-1}}{\left[\frac{\mu\:}{{\alpha\:}^{-1}+\mu\:}\right]}^{\gamma\:}$$

The standard gamma function, denoted by $\:{\Gamma\:}$, plays a role in this context. An additional parameter, often referred to as the ‘ancillary parameter’ $\:\left(\alpha\:\right)$, influences the extent of spread in the predictions. A higher value of $\:\left(\alpha\:\right)$ corresponds to greater data dispersion. When $\:\left(\alpha\:\right)$ is set to 0, the binomial negative model simplifies to the Poisson regression model. Estimating the negative binomial model typically involves the use of the nonlinear maximum likelihood Newton-Raphson algorithm. The moderated multivariable negative binomial analysis provided interaction effects of functional health variables (ADL and IADL) with age at migration on depressive symptoms among older adults. All the models were controlled for selected covariates. The results were presented in the form of an adjusted Incidence rate ratio (IRR) with a 95% confidence interval (CI). Individual weights were used to make the estimates nationally representative. For all the analyses, STATA version 18 has been used^56^.

## Results

Table 1 unveils the sociodemographic characteristics of the study participants. A significant portion of migrants fell within the 45–59 age range (51.27%) and lacked formal education (56.55%). The majority were women (76%), and nearly 28% of migrants were widowed. About 36% of migrants indicated that they were unemployed, with 4.1% living alone. Non-migrants exhibited a higher prevalence of severe food insecurity (6.55%) compared to migrant older adults (5.94%).

Urban residency was more common among migrants (40%) in contrast to non-migrants (26.81%). Migrants also demonstrated a higher prevalence of physical inactivity (35.68%) than non-migrants (37.25%). Additionally, 19% and 10% of migrants reported poor health and difficulties in ADL, respectively. Almost 19% reported IADL issues, and 21% reported experiencing multimorbidity.

**Table 1:** Socioeconomic and demographic profile of the study population in India.

Background characteristics	Non-migrant		Migrant	
	Sample	Percentage	Sample	Percentage
Age				
Middle aged (45–59)	13,980	49.18	18,524	51.27
Young-old (60–69)	7820	29.44	10,149	29.79
Old-old (70–79)	3831	15.36	4602	14.22
Oldest-old (80+)	1379	6.02	1570	4.71
Sex				
Male	19,006	75.22	9795	23.47
Female	8004	24.78	25,050	76.53
Education				
No education	11,625	42.76	17,460	56.55
Primary	5245	19.34	6099	15.90
Secondary	7153	25.24	7789	18.28
Higher	2987	12.66	3497	9.27
Marital status				
Currently married	21,302	79.04	25,043	69.69
Widowed	4588	17.44	8877	27.96
Others	1120	3.52	925	2.35
Living arrangement				
Living with spouse	21,063	78.41	24,456	68.45
Living alone	847	3.01	1294	4.13
Living with others	5100	18.58	9095	27.42
Working status				
Never worked	4110	11.86	12,908	36.54
Retired	7300	28.50	8805	26.24
Currently working	15,600	59.64	13,132	37.23
MPCE quintile				
Poorest	5766	21.81	6544	20.57
Poorer	5598	22.15	6861	20.48
Middle	5425	20.01	7001	20.59
Richer	5284	18.78	7212	19.95
Richest	4937	17.25	7227	18.41
Food insecurity				
No	15,344	56.94	19,081	54.66
Mild	9887	34.53	13,326	37.3
Moderate	488	1.98	749	2.09
Severe	1291	6.55	1689	5.94
Religion				
Hindu	19,344	82.86	26,642	83.66
Muslim	3067	11.02	3472	9.33
Others	4599	6.13	4731	7.11
Caste				
SC/ST	10,412	28.95	11,447	28.01
OBC	10,625	47.46	13,789	46.33
Others	5973	23.59	9609	25.66
Residence				
Rural	19,769	73.19	20,808	59.72
Urban	7241	26.81	14,037	40.28
Physical activity				
Active	16,767	62.75	21,407	64.32
Inactive	10,243	37.25	13,438	35.68
Smoking				
Non-smoker	21,802	79.92	31,625	92.00
Current smoker	5208	20.08	3220	8.00
Alcohol				
Non-heavy drinker	25,438	95.25	33,920	97.92
Heavy episodic drinker	1572	4.75	925	2.08
Self-rated health				
Good	22,904	83.20	28,582	80.80
Poor	4106	16.80	6263	19.20
ADL difficulties				
No	25,332	92.73	31,932	90.20
Yes	1678	7.27	2913	9.80
IADL difficulties				
No	21,914	83.92	26,044	79.42
Yes	5096	20.58	8801	18.66
Multimorbidity				
No	22,762	83.90	27,532	79.42
Yes	4248	16.10	7313	20.58
Cognitive impairment				
No	22,140	89.25	26,890	85.17
Yes	2666	10.75	4681	14.83
Total	27,010	43.67	34,845	56.33

Counts are un-weighted, and percentages are weighted, *MPCE* Monthly per capita consumption expenditure, *SC/ST* Scheduled caste/scheduled tribe, *OBC* Other backward classes, *SRH* Self-Rated Health, *ADL * Activities of daily living, *IADL* Instrumental activities of daily living.

Table 2 presents the average level of depression categorized by migration status. The prevalence of depression increased with age for both migrants and non-migrants. For instance, among migrants, depression was higher among the oldest individuals (80+) at 11.11%, compared to middle-aged individuals^45–59^ at 9.69%. The mean depression scores were elevated for both widowed migrants (10.77%) and non-migrants (10.25%) in comparison to their married counterparts, with scores of 9.59% and 9.21%, respectively. Retired migrants (10.29%) and those belonging to the poorest wealth quintile (10.29%) reported higher depression levels. Additionally, both migrants (12.87%) and non-migrants (12.13%) experiencing severe food insecurity exhibited higher depression levels than their counterparts. Furthermore, individuals who were physically inactive (migrants 10.59%, non-migrants 10.09%), had poor health (migrants 11.63% & non-migrants 11.21%), faced ADL difficulty (migrants: 12.02% & non-migrants: 11.64%), IADL difficulty (migrants: 11.06% & non-migrants: 10.69%), had multimorbidity (migrants: 10.58% & non-migrants: 9.92%) and cognitive impairment (migrants: 14.83% & non-migrants: 10.75%) reported higher mean depression scores compared to their respective counterparts.

**Table 2:** Mean of depressive symptoms stratified by migration status in India.

Background characteristics	Non-migrant			Migrant		
	Mean		Standard error	Mean		Standard error
Age						
Middle-aged (45–59)	9.15		0.03	9.69		0.03
Young-old (60–69)	9.60		0.05	10.05		0.04
Old-old (70–79)	9.80		0.07	10.20		0.06
Oldest-old (80+)	10.15		0.11	11.11		0.12
Sex						
Male	9.34		0.03	9.58		0.04
Female	9.74		0.05	10.05		0.03
Education						
Higher	10.08		0.04	10.45		0.03
No education	9.56		0.06	9.75		0.05
Primary	8.81		0.05	9.29		0.05
Secondary	8.35		0.07	8.44		0.07
Marital status						
Currently married	9.21		0.03	9.59		0.03
Widowed	10.25		0.06	10.77		0.05
Others	10.62		0.13	10.35		0.13
Living arrangement						
Living with spouse	11.38		0.15	11.51		0.12
Living alone	9.20		0.03	9.58		0.03
Living with others	10.13		0.06	10.59		0.04
Working status						
Currently working	9.15		0.03	9.65		0.04
Never worked	9.60		0.06	9.98		0.04
Retired	9.99		0.05	10.29		0.05
MPCE quintile						
Poorest	9.70		0.05	10.29		0.05
Poorer	9.50		0.05	10.04		0.05
Middle	9.30		0.05	9.96		0.05
Richer	9.46		0.06	9.69		0.05
Richest	9.18		0.06	9.67		0.05
Food insecurity						
No	8.70		0.03	9.26		0.03
Mild	10.05		0.04	10.37		0.03
Moderate	11.28		0.19	11.73		0.15
Severe	12.13		0.12	12.87		0.11
Religion						
Others	9.47		0.03	10.3		0.04
Hindu	9.69		0.07	9.95		0.04
Muslim	8.66		0.06	9.53		0.04
Caste						
Others	9.68		0.04	9.93		0.03
SC/ST	9.42		0.04	10.38		0.07
OBC	9.18		0.05	9.46		0.06
Residence						
Rural	9.65		0.03	10.19		0.03
Urban	8.82		0.05	9.48		0.03
Physical activity						
Active	9.06		0.03	9.58		0.03
Inactive	10.09		0.04	10.59		0.04
Smoking						
Non-smoker	9.32		0.03	9.93		0.02
Current smoker	9.92		0.06	10.04		0.07
Alcohol						
Non-heavy drinker	9.43		0.03	9.92		0.02
Heavy episodic drinker	9.59		0.10	10.73		0.15
Self-rated health						
Good	9.08		0.03	9.54		0.02
Poor	11.21		0.07	11.63		0.06
ADL difficulties						
No	9.27		0.02	9.71		0.02
Yes	11.64		0.11	12.02		0.08
IADL difficulties						
No	9.10		0.03	9.46		0.02
Yes	10.69		0.06	11.06		0.05
Multimorbidity						
No	9.35		0.03	9.77		0.02
Yes	9.92		0.06	10.58		0.05
Cognitive impairment						
No	9.24		0.02	9.67		0.02
Yes	10.58		0.07	10.89		0.06
Total	9.44		0.24	9.94		0.22

*MPCE* Monthly per capita consumption expenditure, *SC/ST* Scheduled caste/scheduled tribe, *OBC* Other backward classes, *SRH* Self-Rated Health, *ADL* Activities of daily living, *IADL* Instrumental activities of daily living.

Negative binomial regression was utilized to assess the prevalence of depressive symptoms in middle-aged and older adults based on their migration status, as presented in Table 3. Controlling socioeconomic and demographic factors revealed a noteworthy increase in the occurrence of depressive symptoms with advancing age at migration among migrant adults. Specifically, individuals migrating at ages 40–59 exhibited a 1.05 times higher incidence of depressive symptoms (IRR: 1.05; 95% CI: 1.02, 1.09) who migrated before the age of 20 (IRR: 1.03; 95% CI: 1.02, 1.05) compared to non-migrants. Additionally, the prevalence of depressive symptoms was 1.12 times higher among migrants with no formal education (IRR: 1.12; 95% CI: 1.09, 1.15) in comparison to highly educated migrants. Furthermore, individuals living alone (IRR: 1.05; 95% CI: 1.01, 1.10), experiencing severe food insecurity (IRR: 1.29; 95% CI: 1.26, 1.32), reporting poor health conditions (IRR: 1.13; 95% CI: 1.11, 1.15), facing ADL challenges (IRR: 1.10; 95% CI: 1.07, 1.12), and IADL difficulties (IRR: 1.06; 95% CI: 1.04, 1.08) exhibited higher incidences of depressive symptoms.

**Table 3:** Negative binomial regression estimates for depressive symptoms among middle-aged and older adults by migration status in India.

Background characteristics	Model 1		Model 2		Model 3	
	IRR	95% CI	IRR	95% CI	IRR	95% CI
Age at migration						
Non-migrant	Ref.		Ref.		Ref.	
< 20	1.02**	[0.97, 1.08]	1.02***	[1.01, 1.04]	1.03***	[1.02, 1.05]
20–40	1.02*	[1.01, 1.04]	1.02	[0.99, 1.08]	1.04***	[1.01, 1.05]
40–59	1.04*	[1.01, 1.08]	1.03	[1.01, 1.07]	1.05***	[1.02, 1.09]
> 60	1.06**	[1.00, 1.11]	1.01	[0.94, 1.05]	1.01	[0.92, 1.07]
/lnalpha	−2.61***	[−2.66, −2.55]	−2.57***	[−2.62, −2.51]	−2.94***	[−3.01, −2.87]
Alpha	0.07***	[0.06, 0.08]	0.08***	[0.07, 0.09]	0.05***	[0.04, 0.06]
Observation (n)						

Model 1 is unadjusted, model 2 is adjusted only age and sex of the respondents and model 3 is adjusted for age, sex, education, living arrangement, food insecurity, religion, caste, residence physical activity, smoking, alcohol, Self-Rated Health, difficulties in ADL, difficulties in IADL, multimorbidity, cognitive impairment. *Ref *Reference, *CI* Confidence interval, *MPCE* Monthly per capita consumption expenditure,* SC/ST* Scheduled caste/scheduled tribe, *OBC* Other backward classes, SRH: Self-Rated Health, *ADL* Activities of daily living, *IADL* Instrumental activities of daily living. Alpha is the estimate of the dispersion parameter. The dispersion parameter alpha can be obtained by exponentiating/lnalpha. If the dispersion parameter equals zero, the model reduces to the simpler Poisson model. If the dispersion parameter (alpha) is significantly greater than zero than the data are over dispersed and are better estimated using a negative binomial model than a Poisson model.

**Fig. 3:**
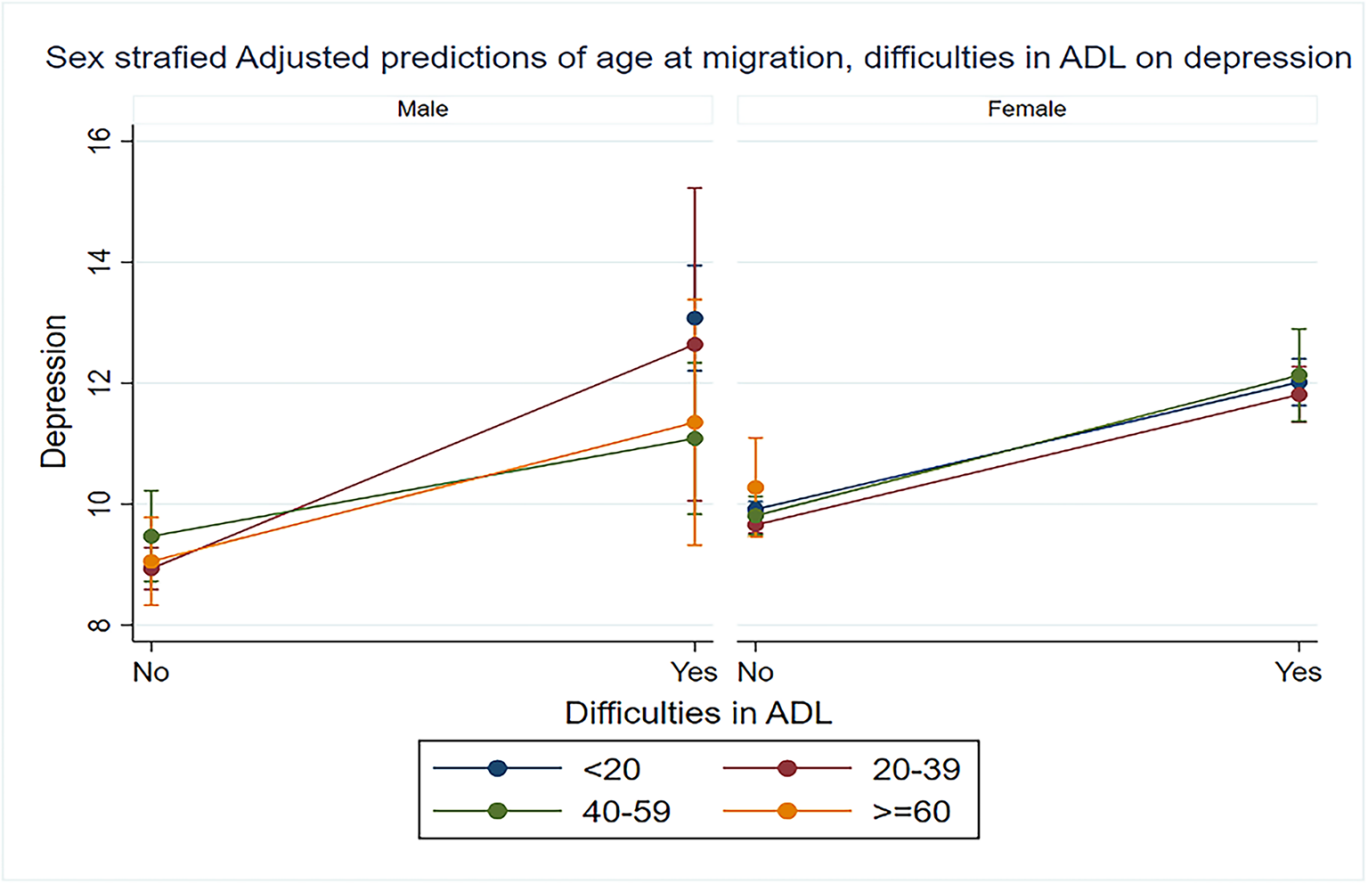
Margins plot of the sex stratified interaction of age at migration with difficulties in ADL on depression among older adults.

Figure 3 illustrates how the combination of age at migration and difficulties in ADL interacts with depression among older adults, considering sex differences. Both male and female migrants facing challenges in ADL reported significantly higher levels of depressive symptoms. Notably, male migrants who encountered difficulties in performing ADL activities and migrated during their Middle-ages were found to be more prone to experiencing depression. Conversely, female migrants who migrated at a younger age and faced limitations in ADL were also more likely to suffer from depression.

Table 4 shows the interaction between challenges in ADL and the age at migration concerning depression among older adults in India. After controlling socioeconomic and demographic factors, it was observed that depression levels escalated with an increase in the age at migration. The likelihood of experiencing depression was 1.12 times higher (IRR: 1.12; 95% CI: 1.07, 1.18) for individuals who migrated before the age of 20. The risk of being depressed was 1.13 times and 1.15 times higher for those migrating between the ages of 20–39 and 40–59, compared to non-migrants.

**Table 4:** Interaction of difficulties in ADL and age at migration on depression among older adults in India.

Background characteristics	Unadjusted		Adjusted	
	IRR	95% CI	IRR	95% CI
ADL X Age at migration				
No X Non-migrant	Ref.		Ref.	
No X < 20	1.02***	[1.00, 1.04]	1.03***	[1.01, 1.05]
No X 20–39	1.02*	[1.00, 1.05]	1.04***	[1.01, 1.07]
No X 40–59	1.04	[1.00, 1.08]	1.06**	[1.02, 1.11]
No X > 60	1.04***	[0.98, 1.11]	1.04	[0.97, 1.18]
Yes X < 20	1.20***	[1.17, 1.25]	1.12***	[1.07, 1.18]
Yes X 20–39	1.27***	[1.21, 1.37]	1.13***	[1.08, 1.19]
Yes X 40–59	1.29***	[1.20, 1.35]	1.15***	[1.08, 1.22]
Yes X > 60	1.25	[1.09, 1.33]	1.16	[0.99, 1.36]
Yes X Non-migration	1.26***	[1.22, 1.30]	1.11**	[1.07, 1.15]
/lnalpha	−2.63	[−2.68, −2.56]	−2.93	[−3.01, 2.86]
alpha	0.07***	[0.06, 0.08]	0.05***	[0.04, 0.06]
Observation (n)	61,855		61,855	

*ADL* Activities of daily living, Model 2 (Adjusted) is adjusted for all sociodemographic, behavioural and health-related factors.

Furthermore, the association between depression and the interaction of IADL and age at migration is illustrated in Table 5. The findings from the table illustrated that individuals who migrated at any age of their life and had problems in performing IADL activities were likely to face depression than their non-migrant counterparts. However, the findings from the interaction model were not significant enough to make any distinction from the non-migrants with or without IADL limitation in depressive symptoms.

**Table 5:** Interaction of difficulties in IADL and age at migration on depression among older adults in India.

Background characteristics	Unadjusted		Adjusted	
	IRR	95% CI	IRR	95% CI
IADL X Age at migration				
No X Non-migrant	Ref.		Ref.	
No X < 20	1.02***	[1.04, 1.08]	1.03***	[1.01, 1.06]
No X 20–39	1.01	[0.97, 1.04]	1.04**	[1.02, 1.07]
No X 40–59	1.03*	[1.00, 1.06]	1.04**	[1.01, 1.08]
No X > 60	1.03	[0.95, 1.11]	1.03	[0.97, 1.10]
Yes X < 20	1.21***	[1.19, 1.24]	1.03	[0.98, 1.08]
Yes X 20–39	1.22***	[1.18, 1.25]	1.06	[0.99, 1.12]
Yes X 40–59	1.23***	[1.14, 1.33]	1.05	[0.97, 1.15]
Yes X > 60	1.23***	[1.17, 1.27]	1.03	[0.93, 1.12]
Yes X Non-migration	1.17***	[1.14, 1.22]	1.02	[0.97, 1.08]
/lnalpha	−2.63	[−2.69, −2.57]	−2.94	[−3.02 , −2.90]
alpha	0.07***	[0.05, 0.09]	0.05***	[0.04, 0.06]
Observation (n)	61,855		61,855	

* IADL* Instrumental Activities of daily living, Model 2 (Adjusted) is adjusted for all sociodemographic, behavioural and health-related factors.

**Fig. 4:**
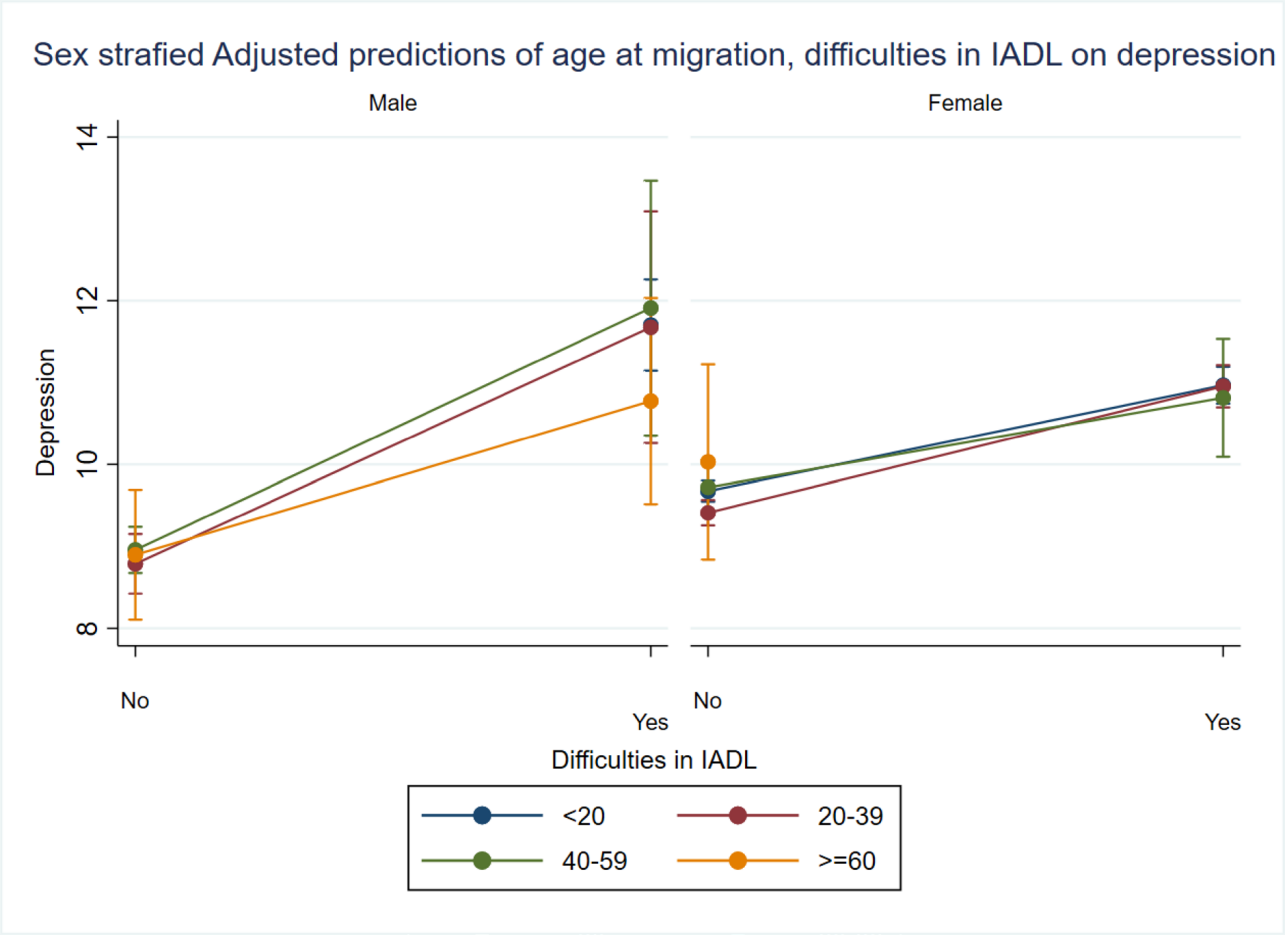
Margins plot of the sex stratified interaction of age at migration with difficulties in IADL on depression among older adults.

In Fig. 4, it is evident that male migrants who experienced difficulties in IADL and migrated before the age of 20 are more likely to suffer from depression. On the other hand, female migrants with IADL impairments are likely to exhibit a similar depressive symptom irrespective of their age at the time of migration to destination areas.

## Discussion

The current study examined how functional limitation influences the association between age at migration and depressive symptoms among middle-aged and older migrants. Prior studies have shown that functional limitations have important implications for the well-being of older migrants^29^. Although depression in late life is a widespread concern, researchers have not developed a nuanced understanding of how age at migration and functional limitation influences the effect of depressive symptoms among middle-aged and older migrants. The current results indicate that age at migration and functional limitation can matter in such associations. In consideration of the first hypothesis addressing whether migration at later ages is linked to heightened depression, our study yields insightful results. The results indicate a significant association between the age at migration in the later stages of life and elevated depressive symptoms. This is perhaps because the age at which migration occurs influences migrants’ capacity to adapt to a new environment, encompassing language acquisition and social engagement opportunities^57^. These variations in migration motivations and experiences are instrumental in shaping the impact of migration-related stress and depression on health outcomes, as highlighted by^58^. Factors such as encounters with change, loss, perceived discrimination, and social marginalization have been associated with the manifestation of depressive symptoms^59^. Moreover, depression has a cascading effect on various facets of health, leading to declines in physical, mental, functional, and overall well-being^60^. Consequently, it is unsurprising that depression emerges as a significant contributor to morbidity among older migrants. Mills^61^ conducted a study indicating that individuals who migrate to the United States, whether in later life or during childhood, face an elevated risk of depression compared to their native-born counterparts. This aligns with previous research, such as that of^30^, which demonstrated that migrants arriving at older ages tend to exhibit more depressive symptoms. Adapting to a new culture, particularly for older migrants who have deep roots in their own cultural backgrounds, can be quite challenging, especially when there is a strong emphasis on family ties. It is crucial to examine how older migrants can successfully navigate and adjust to their new family environments, considering the potential complexities that may arise. The need to understand this aspect is underscored by the fact that this population is rapidly expanding. A study by Honkaniemi^47^ in Europe found that the marginal effects of migration status on the probability of experiencing psychological distress tended to increase with age at migration among migrants.

In reference to our second hypothesis, which delves into the role of physical limitations in moderating the association between age at migration and depressive symptoms among middle-aged and older adults in India, the results indicated that ADL limitations significantly mediated the association between age at immigration and depressive symptoms, accounting for known confounding factors. In Indian culture, disability and functional impairments frequently result in social seclusion and the imposition of stigmas. People with disabilities may encounter difficulties in engaging in social activities due to physical obstacles or societal biases^62^. The ensuing isolation can foster feelings of loneliness and exclusion, triggering or worsening depression. This finding aligns with findings from prior studies examining the association between functional limitations and depression. For example, an investigation carried out in India by^62^ found an association between ADL and IADL problems and heightened levels of depression among the elderly. Similarly, an analysis across 11 European countries showed a heightened prevalence of depression among older adults with disabilities compared to those without disabilities^63^. Another cross-sectional study involving low-income African Americans, using functional limitation as a measure of dependence, identified depression, as a mediator between pain intensity and functional limitations assessed by ADL^64^. A longitudinal study in Beijing also established that the development of physical limitations significantly increases the risk of depression^65^.

Our study further indicates that older adults with higher education face a lower likelihood of experiencing depression. This aligns with previous research consistently demonstrating a negative connection between educational achievement and depression^62,66^. Those with advanced education levels often display increased awareness and skills in addressing psychological concerns, potentially lessening the likelihood of developing such conditions^67^. Additionally, higher educational attainment correlates with a diminished risk of depression, as individuals with more education tend to be more conscious of available health services, resulting in improved utilization of healthcare resources^66^. Middle-aged and older Individuals who live with a spouse benefit from consistent companionship, emotional support, and practical assistance^68,69^. Marriage offers these advantages and expands opportunities for social integration^70^. Unsurprisingly, middle-aged and older migrants residing with a spouse tend to experience better mental health compared to those living alone or with other family members^69^. In Indian context, the disruption of traditional values and family support systems among migrants can exacerbate mental health challenges, heightening the stress experienced and leading to a greater sense of social isolation^71^. Older adults living alone often experience a decline in mental well-being, commonly attributed to social isolation, a connection highlighted by our study. Numerous other studies have similarly identified and affirmed this association^72,73^.

We found a notable association between mental health and food insecurity in middle-aged and older adults of both genders. It was observed that as food insecurity levels rose, so did the likelihood of experiencing depression. This finding is consistent with research conducted in both developed^74^ and developing countries^75,76^, indicating an association between food insecurity and an elevated risk of depression. Understanding the intricate relationship between food insecurity and depression remains a complex challenge due to the multifactorial nature of both phenomena^77^. Existing literature indicates that food insecurity may function as an environmental stressor, potentially contributing to late-life depressive disorders^78,79^. The psychological pathways linking food insecurity to depression involve anxiety and life stress, stemming from concerns about social standing and financial constraints^53,78^ Self-rated health is a critically important measure of how diseases impact an individual’s overall well-being among elderly migrants^29^. Our research established the positive association between self-perceived health and depressive symptoms among middle-aged and older migrants. A multitude of research also suggests that the degree to which depressive symptoms occur is influenced by an individual’s perception of their health^80,81^. In addition to poor-SRH, depressive symptoms are often associated with a person’s chronic illnesses^82^. A current study by Saha et al.^71^ based on older adults in India shows that the level of depression was significantly higher among multimorbid patients than others. Our research also confirms that middle-aged and older migrants with multimorbidity face an increased risk of depression compared to those without such health conditions. This aligns with earlier studies that observed a co-occurrence of multimorbidity and depression in elderly migrants, indicating a heightened susceptibility to depression and other mental health issues^83^. The elevated vulnerability to mental health challenges in individuals with multimorbidity may arise from heightened physical discomfort and increased psychological burden associated with managing multiple health conditions. As argued by Ito^84^ illustrated financial costs related to the treatment of these chronic illnesses could also contribute to this situation. Managing various chronic conditions in older adults in a new place intensifies the overall disease burden, leading to a higher incidence of psychological distress, especially for migrants^83^. Our study also reveals the association between smoking and depression, shedding light on a significant link of depression among older adults who were current smokers. The evidence presented supports the notion that individuals who smoke face an increased susceptibility to depression. This association aligns with existing literature highlighting the adverse impact of smoking on mental health^85–87^.

While this study offers valuable insights into the relationship between age at migration and mental health, a few limitations must be acknowledged. First, LASI did not provide data on migration motives, which could significantly influence depression. Voluntary and involuntary migrants may experience and perceive depression differently, with the latter potentially facing higher stress and trauma. Future research should explore migration motives to better understand their role in mental health outcomes. Second, India’s cultural and linguistic diversity means migration to different regions may have varying psychological impacts, shaped by factors like language barriers and social integration challenges. Regional determinants, such as discrimination or social support, could further moderate these effects. Future studies should examine regional variations to provide a more nuanced understanding of migration’s mental health implications. Third, reliance on self-reported data introduces potential recall and social desirability biases, which may distort the observed association between age at migration and depressive symptoms. For instance, older migrants might underreport symptoms due to stigma, while younger migrants might overreport them due to greater mental health awareness. Future research could use mixed-method approaches, combining self-reports with clinical assessments or qualitative interviews, to validate findings and reduce bias. Fourth the study did not contextualize differences between origin and destination places, which could influence the strength of the observed association. Finally, the cross-sectional design limits our ability to infer causality or capture changes over time. Migrants who moved earlier in life may have better baseline cognitive health than those who migrated later. To better understand how factors before and after migration, along with multiple relocations throughout one’s life, impact long-term mental health, there is a need for longitudinal studies that follow migrants through various life stages.

## Conclusion and policy implications

This study represents the initial attempt to investigate the relationship between age at migration and cognitive health among a large sample of older Indian migrants. To the best of the authors’ knowledge, it is the first study of its kind. Our results align closely with findings from global studies^30,47,88^, indicating that migrating later in life could significantly impact factors related to cognitive function in later years, thereby elevating the risk of psychological distress among migrants who migrated later in life. The implications of these findings are significant for both research and practical applications. Highlighting the diversity among migrants concerning the age of migration and depression, this study aids in recognizing specific vulnerable segments among older immigrants. To mitigate the heightened risk of depression among older migrants with functional limitations, targeted mental health support and improved healthcare accessibility are essential. Establishing community-based mental health programs can provide culturally sensitive counseling and interventions to address migration-related stress and isolation. Additionally, dedicated healthcare units in migrant-dense areas should be developed to offer comprehensive medical and psychological support. These units can provide early screening, subsidized treatments, and rehabilitation services for individuals with ADL and IADL limitations, ensuring timely care. Mobile health clinics should also be deployed to extend healthcare access, particularly for economically disadvantaged migrant populations. Existing policies require urgent modifications to better serve migrant populations. *The National Program for Health Care of the Elderly* should be expanded to offer portable geriatric care for old age migrants, integrating services with the *Ayushman Bharat Digital Mission* to maintain continuity of care across states. The *Pradhan Mantri Bhartiya Janaushadhi Pariyojana* must establish low-cost medicine outlets near migrant-dense regions to ensure affordable access to essential medicines. Beyond healthcare, broader welfare initiatives must also be adapted for migrants. Additionally, *Fit India* should include community-based exercise programs in migrant settlement areas.

## Supplementary Information

Below is the link to the electronic supplementary material.

Supplementary Material 1

## Data Availability

The data that support the findings of this study are available from https://g2aging.org/but restrictions apply to the availability of these data, which were used under license for the current study, and so are not publicly available. Data are however available from the corresponding author upon reasonable request and with permission of https://www.iipsindia.ac.in/.
