# Supplementary material for: Later-life migration and depression among older adults in India: examining the role of functional limitations
Source: Sci Rep. 2025 Oct 22;15:36832. doi: 10.1038/s41598-025-14489-9 (PMC12546585; doi:10.1038/s41598-025-14489-9)
Supplement: Supplementary file 1 — Supplementary Material 1 [file 41598_2025_14489_MOESM1_ESM.docx]

**Supplementary File**

**Table A1** Details of covariates included in the study

| **Variables** | **Categories** | **Description of the category** |
| --- | --- | --- |
| **Age** | Middle-aged (45-59) | Age of the respondents were categorized into “middle-aged (45-59 years)” "young-old (60-69 years)," "old-old (70-79 years)," and "oldest-old (80 years & above)". |
|  | Young-old (60-69 years) |  |
|  | old-old (70-79 years) |  |
|  | oldest-old (80 years & above) |  |
| **Sex** | Male | Sex of the respondent was available in male-female categories. |
|  | Female |  |
| **Place of residence** | Rural | Place of residence (rural/urban) was determined according to the administrative division of India followed in Census of India, 2011. Households in urban areas included those in towns, wards and Census Enumeration Blocks whereas, households in rural areas include those in villages (size varies from 0-10,000 population. |
|  | Urban |  |
| **Education** | No education/ primary not completed | There were four categories for educational status: “No education”, “Primary”, “Secondary”, and “Higher”. |
|  | Primary |  |
|  | Higher |  |
|  | Secondary |  |
| **Marital status** | Currently married | Marital status was categorized as Married, widowed and others (living alone, separated). |
|  | Widowed |  |
|  | Others |  |
| **Working status** | Working | Furthermore, working status was recoded as "Working," "Retired," and "Not working" in this study. |
|  | Retired |  |
|  | Not working |  |
| **MPCE quantile** | Poorest | Utilizing household consumption data, we evaluated the Monthly Per Capita Consumption Expenditure (MPCE) quintile. The surveyed sample households responded to sets of 11 and 29 questions concerning spending on food and non-food items, respectively. Food expenditures were recorded over a seven-day reference period, while non-food expenditures were collected for 30-day and 365-day reference periods. Standardization of expenses for both food and non-food items occurred using a 30-day reference period. The MPCE, serving as a summary indicator of consumption, was calculated and categorized into five quintiles, ranging from the poorest to the richest. |
|  | Poor |  |
|  | Middle |  |
|  | Richer |  |
|  | Richest |  |
| **Food insecurity** | No | Food security was assessed through four questions, leading to the categorization of respondents based on the severity of scarcity of food. Those who responded affirmatively to the survey question, "Was hungry but did not eat or did not eat for a whole day because there was not enough food in their household," were categorized as experiencing severe food insecurity. Respondents indicating that they "reduced the size of their meals or skipped meals because there was not enough food in the household" were classified as having moderate food insecurity. Individuals reporting that they "did not eat enough food of their choice (excluding fasting/food-related restrictions)" were labeled as experiencing mild food insecurity. Those who did not face any such scarcity were classified as persons with "no food insecurity." |
|  | Mild |  |
|  | Moderate |  |
|  | Severe |  |
| **Religion** | Hindu | Religion of the respondents was categorized into Hindu, Muslim, Christian, and others. |
|  | Muslim |  |
|  | Christian |  |
|  | Others |  |
| **Caste** | Scheduled castes (SC) | Caste was categorized into SC, ST, OBC, and others. SC and ST represent the most economically and socially disadvantaged groups in India. In the Hindu caste system, the SC encompasses a population segment that experiences social isolation and holds a lower economic position. Individuals considered "educationally, economically, and socially backward" fall under the OBC category. Traditionally, in the caste hierarchy, OBC is perceived to be above the most disadvantaged communities but still at a lower tier. The "other" caste category comprises those with higher social standings. |
|  | Scheduled Tribes (ST) |  |
|  | Other backward classes (OBC) |  |
|  | Others |  |
| **Self-rated health** | Poor | Self-rated health was assessed through a single question, inquiring about one's overall health status. Respondents could choose from five response categories: "Very poor," "Poor," "Fair," "Good," and "Very good." For this study we clubbed very poor and poor category and formed “poor” and others are as “good”. |
|  | Good |  |
| **Physical activity status** | Yes | Those engaged in either moderate physical activity (at least 150 minutes throughout the week) or vigorous physical activity (at least 75 minutes throughout the week), or an equivalent combination of both, were categorized as physically active. Physically inactive respondents were those not engaged in any type of moderate or vigorous physical activity throughout the week. |
|  | No |  |
| **Tobacco consumption** | Yes | The definition of current smokers includes individuals who consume smoking tobacco exclusively; otherwise, they are classified as nonsmokers. |
|  | No |  |
| **Alcohol consumption** | Yes | Those who consumed more than 5 drinks on any occasion in the past 30 days were classified as heavy drinkers, while others were categorized as non-heavy drinkers. |
|  | No |  |
| **Multimorbidity** | Yes | Multimorbidity is defined as the coexistence of two or more chronic diseases. The LASI survey encompassed nine specific chronic diseases, including (1) hypertension or high blood pressure, (2) diabetes, (3) cancer, (4) any chronic lung disease such as asthma, (5) chronic heart diseases, (6) stroke, (7) bone/joint disease, (8) any neurological or psychiatric diseases, and (9) high cholesterol. |
|  | No |  |
| **Cognitive impairment** | Yes | Cognitive functions in the study were evaluated across five key areas: memory, orientation, arithmetic abilities, executive functions, and the ability to name objects. These assessments were adapted from the methodologies used in the Health and Retirement Study. In detail, memory was assessed by tasks such as immediate and delayed word recall, scoring up to 10 points each. Orientation was determined by participants’ ability to recognize time and place, each contributing up to 4 points. Arithmetic capabilities were tested using methods like backward counting and serial subtraction tasks, along with other computational tests, contributing up to a total of 9 points. Executive functions were examined through tasks like paper folding and drawing pentagons, scoring up to 4 points in total. Additionally, object naming was included, where participants could score up to 2 points. A comprehensive score ranging from 0 to 43 was calculated from these individual tests, with higher scores indicating better cognitive health. Cognitive impairment was specifically identified in those falling within the lowest 10th percentile. Participants needing assistance during the cognitive tests were not included in the analysis. |
|  | No |  |

**Table A2** Questions to assess depressive symptoms (CES-D)

| 1 | How often did you have trouble concentrating? |
| --- | --- |
| 2 | How often did you feel depressed? |
| 3 | How often did you feel tired or low in energy? |
| 4 | How often were you afraid of something? |
| 5 | How often did you feel you were overall satisfied? |
| 6 | How often did you feel alone? |
| 7 | How often were you bothered by things that don’t usually bother you? |
| 8 | How often did you feel that everything you did was an effort? |
| 9 | How often did you feel hopeful about the future? |
| 10 | How often did you feel happy? |

**TABLE A3** Questions to assess ADL difficulty

| 1 | Dressing, including putting on chappals, shoes, etc. |
| --- | --- |
| 2 | Walking across a room |
| 3 | Bathing |
| 4 | Eating, difficulties |
| 5 | Getting in or out of bed |
| 6 | Using the toilet, including getting up and down |

**TABLE A4** Questions to assess IADL difficulty

| 1 | Preparing a hot meal (cooking and serving) |
| --- | --- |
| 2 | Shopping for groceries |
| 3 | Making telephone calls |
| 4 | Taking medications |
| 5 | Doing work around the house or garden |
| 6 | Managing money, such as paying bills and keeping track of expenses |
| 7 | Getting around or finding address in unfamiliar place |
